# The Dual Role of the Immune Response in Reproductive Organs During Zika Virus Infection

**DOI:** 10.3389/fimmu.2019.01617

**Published:** 2019-07-11

**Authors:** Haruki Arévalo Romero, Tania A. Vargas Pavía, Manuel A. Velázquez Cervantes, Arturo Flores Pliego, Addy C. Helguera Repetto, Moises León Juárez

**Affiliations:** ^1^Laboratory of Immunology and Molecular Microbiology, Multidisciplinary Academic Division of Jalpa de Méndez, Department of Genomics, University Juárez Autonomous of Tabasco, Jalpa de Méndez, Mexico; ^2^Laboratory of Perinatal Virology, Department of Immuno-Biochemistry, National Institution of Perinatology “Isidro Espinosa de los Reyes”, Mexico City, Mexico

**Keywords:** Zika virus, sexual transmission, immune response, reproduction, interferon

## Abstract

Zika virus is a mosquito-borne viral disease that emerged as a significant health problem in the Americas after an epidemic in 2015. Especially concerning are cases where Zika is linked to the development of brain abnormalities in newborns. Unlike other flaviviruses, Zika can be transmitted sexually, increasing the potential for intraspecies infection. Several reports show that the virus can persist for months in the testis of males after clearance of viremia, and that females are highly susceptible to infection via sexual transmission. The most common route of sexual transmission is male-to-female, which suggests that the mechanism driving persistence of Zika in the testis is essential for dissemination. The immune system plays an essential role in Zika infection. In females, a robust response inhibits the virus to control the infection. In males, however, the immunological response to Zika infection correlates with viral persistence. Thus, the immune system may have a dual role in sexually transmitted pathogenesis. The mechanism by which the immune system allows the virus to enter an immune-privileged site while continuing to disseminate is unclear. In this mini-review, we highlight advances in our knowledge of sexually transmitted Zika virus pathogenesis and the possible mechanisms mounted by the immune system that control or exacerbate the infection.

## Introduction

Zika virus (ZIKV) is a health risk worldwide that is disseminated primarily by insect vectors. Tracking the outbreak of Zika in the Americas in 2015 showed that the virus had been circulating in several countries in Africa and Asia. It is primarily transmitted through *Aedes aegypti* mosquito bites (1, 2). Infection may cause several health complications including microcephaly and congenital Zika syndrome that are associated with vertical transmission (3). This led the World Health Organization to declare the ZIKV outbreak a public health emergency of international concern in 2016 (4). This problem became even more relevant when it was shown that transmission of the pathogen was not exclusive to mosquito bites, similar to that for other flaviviruses. Clinical evidence shows that ZIKV has the potential for human-to-human transmission through sexual routes, including male-to-female, female-to-male, and male-to-male transmission (5–7).

Additionally, ZIKV infiltrates several biological fluids including breastmilk, urine, tears, and saliva (8–10). Thus, it is possible that ZIKV can spread via multiple routes of infection. Several reports suggest that tropism of ZIKV to sexual organs could be a bridge for sexual transmission similar to that employed by human immunodeficiency virus (HIV-1) or human papilloma virus (HPV) (11, 12). Interestingly, the immune response in the male and female reproductive organs could play a role in the spread, persistence, and clearance of ZIKV infection. In this mini-review, we collect the current evidence supporting a dual role of the immune response in the reproductive system organs during ZIKV infection as a promoter of viral dissemination in males and having protective roles in females.

### Clinical Reports

Foy et al. reported for the first time that ZIKV might be sexually transmissible. The virus was identified in a woman that had not traveled to an area endemic for ZIKV transmission. She developed symptoms of the disease after sexual contact with her husband in the days after he returned from a trip to Senegal (13). Since this initial finding, several studies have been published indicating sexual transmission of ZIKV in other areas not endemic for mosquito transmission, such as Europe and North America (14, 15). Additionally, these reports show that male-to-female transmission is more frequent than female-to-male transmission (16–18). Interestingly, studies that have reported shedding of ZIKV in genital fluids to suggest a more robust presence in semen, compared to that in vaginal fluids, and a clear persistence of ZIKV for at least 188 days following symptom onset (19). Moreover, the only study focused on detecting the ZIKV in vaginal secretions showed that the virus could persist for 60–180 days after the onset of symptoms (20). However, the assays determining the ZIKV viability were not conducted, in contrast to the studies on semen samples that determined the production of infective virus in Vero cells (21). Additionally, a similar behavior was observed in studies with other viral sexual transmission such as Hepatitis C, Ebola, HIV-1, and Herpes identifying high viral loads in seminal fluid samples (22–25). The studies also showed greater transmission from male-to-female and the viral load was evaluated in vaginal fluids, but the test to detect viral viability in these samples was not performed. Altogether, these data suggest that cells residing in the male reproductive system are highly susceptible to viral infection or that the female reproductive system presents a more effective defense against the pathogen. These hypotheses need to be addressed adequately through further research.

### Tropism of ZIKV to Sexual Organs

The male reproductive tract (MRT) constitutes the testis, epididymis, deferens tubules, prostate, seminal vesicle, bulbourethral glands, and penile urethra. These structures are required for the production, maturation, and protection of spermatozoa from immunoreaction and infectious agents (26, 27). The female reproductive tract (FRT) is organized into two functional compartments associated with fetal development (upper FRT) and the external environment (lower FRT). The vagina and ectocervix constitute the lower FRT and are organized by a keratinized stratified squamous epithelium. The endocervix, uterus, and fallopian tubes comprise the upper FRT and consist of a single layer of columnar epithelium (28). Implementation of animal models has been essential to identify the cellular targets of ZIKV within reproductive tracts (29). Results from these models have shown that ZIKV has high tropism in the MRT, infecting cell types that include spermatogonia, primary spermatocytes, Sertoli cells, peritubular myoid cells, Leydig cells, and epithelial cells of the lumen (30). In contrast, in human testis *ex vivo*, ZIKV can infect and replicate in a range of somatic and germ cells, such as testicular macrophages, peritubular cells, Leydig, and Sertoli cells. However, the infection was weaker in human cells than that observed in the mouse model (31). Finally, ZIKV was also found in the spermatozoa from ZIKV-infected men, suggesting that ZIKV could infect the zygote and cause fetal congenital diseases. We propose that the immune response could help ZIKV gain access to spermatozoa in the MRT (32).

In the case of cells within the FRT, few reports have evaluated their susceptibility to ZIKV infection. The experimental evidence from animal model mosquito bite inoculates suggests that ZIKV is present in reproductive tract tissues such as the cervix and vagina (33). Additionally, studies on immunocompetent mice showed that vaginal inoculation of ZIKV lead to productive replication of the virus within the vaginal mucosa and ovarian follicles. Additionally, ZIKV replication in the vaginal tissue of a pregnant mouse was followed by infection of the fetal brain, suggesting vertical transmission (34). Cellular models *in vitro* have shown that human endometrial stromal cells are permissive to ZIKV infection and replication. Interestingly, *in vitro* decidualization of this cellular model increased the replication of ZIKV, suggesting that endometrial cells have an important role during sexual transmission of ZIKV. They might provide a viral reservoir for infection of cells at the maternal-fetal interface during pregnancy (35). Finally, current studies that focus on the use of human tissues or primary cultures of FRT cells need to be performed to evaluate if the behavior of ZIKV infection in animal models is similar to that in humans. The establishment of vaginal and cervical epithelial cells could be an exciting model as they have been used for the study of HIV-1 and Herpes simplex virus (36, 37).

### Role of the Immune System in Sexual Transmission of ZIKV

Viral evolution requires development of a mechanism to evade the host immune response and manipulate cells for their own replication, allowing it to spread to other cells (38). ZIKV evades the immune system (IS) by regulating the type I interferon response with its encoded NS5 protein (39). Mice lacking components of IFN pathway are more susceptible to ZIKV infection, highlighting the importance of the IS for the control of the virus (40).

Among flaviviruses, sexual transmission is unique to ZIKV, marking an unusual potential for human-to-human transmission. The immune privileged nature of the testis protects germ cells from immune attack that occurs during infection. While clinical reports clearly identified persistence of viral RNA in seminal fluid, little is known about how ZIKV passes from blood to the MRT, nor how it persists in the testis. The mechanism seems to involve different cell types, including immune system cells, for transport to target tissues. For example, ZIKV was preferentially found in the cell fraction of blood samples from humans exposed to an endemic area, suggesting that ZIKV could use this mechanism for transport before crossing tissue barriers in the male body (41, 42).

ZIKV seems uses a conserved arboviral strategy for primary infection. Semliki Forest virus (SFV) is an alphavirus that is a close relative to the Chikungunya virus. It was shown that a cutaneous innate immune response involuntarily facilitated viral infection. The mechanism involved neutrophil-dependent inflammation at mosquito bite sites that promoted the recruitment of myeloid cells that are permissive to the virus. These then released new infectious viral particles targeting new cellular targets (43). We hypothesize that the same mechanism may be used by ZIKV to spread its infection. In support of this possibility, human *in vitro*-generated dendritic cells (DCs) are permissive to ZIKV infection (44). Additionally, using a pigtail macaque model it was shown that male animals were more susceptible to ZIKV persistence in peripheral lymph nodes and dissemination to mucosal tissues. The mechanism involves two steps: an inflammatory response that follows ZIKV infection that drives the recruitment of innate cells as early targets of the virus. The infected cells are myeloid DCs, non-classic monocytes, and natural killer (NK) cells. Unlike SFV, the recruitment of cells is driven by plasmocytoid DCs in a MCP-1 dependent manner, which also supports the dissemination and persistence of ZIKV (45). Altogether, these data show that IS inadvertently plays a role in viral tropism, persistence, and pathogenesis of ZIKV.

In general, we consider that there are two routes to reach spermatids in the seminiferous tubules. One is that ZIKV uses the IS to reach the testis and manipulate the antiviral mechanism to disrupt the blood testicular barrier (BTB) junctions. The other possibility is that ZIKV proteins directly destroy the junctions to cross the barrier. We believe these processes are not exclusive, as both are supported by evidence in the literature (45–47). Once ZIKV reaches the testicles, we assume that the virus gains access to germinal cells by three mechanisms. (1) ZIKV can infect or alter the endothelium and pass to the interstitial space, (2) infection of different cells elicits disruption of junctional adhesions in a manner that is dependent on IS antiviral activity or a direct function of ZIKV protein, and (3) direct transmigration of infected immune cells from the blood to the seminiferous tubules.

A recent study supported endothelial passage, showing that ZIKV NS1 protein bound specifically to endothelial cells including human umbilical vein endothelial cells (HUVECs) and human brain microvascular endothelial cells (HBMECs), causing a decrease in their permeability. The mechanism implies degradation of the endothelial glycocalyx layer by the activation of sialidase and cathepsin L-heparanase both *in vitro* and *in vivo* (48). Another piece of evidence is indirect but concordant with endothelial passage. ZIKV could persistently infect and replicates in primary HBMECs. Moreover, ZIKV was delivered from both apical and basolateral surfaces suggesting a direct mechanism to cross the blood brain barrier (49). In addition, it was shown that the Sertoli cells that constitute the BTB were highly susceptible to ZIKV infection suggesting that ZIKV may be able to utilize this crossing mechanism directly (46). Finally, a study using the bluetongue virus in a ram model showed testicular damage due to viral replication in the endothelial cells of the peritubular area in the testis, resulting in the destruction of the Sertoli cell barrier associated with type I interferon response raising the possibility that ZIKV may use a similar mechanism of this related arbovirus in humans (50). Overall, these studies suggest that ZIKV can infect and alter endothelial barriers to facilitate virus dissemination into target organs facing other target cells, such as Sertoli cells, and macrophages in the interstitial space. In parallel, the extravasation of fluids can induce inflammation that also helps the virus to disrupt other barriers, such as BTB, to enhance viral infection. However, studies describing this mechanism in human or mouse endothelial testicular cells are lacking and need to be tested.

For the second mechanism, using an *in vitro* BTB model with human Sertoli cells it was observed that ZIKV could infect these cells for a long term without induction of cell death, and that Sertoli cells can mount a robust antiviral response. The virus did not directly alter tight or adherent junctions, but induced the expression of VCAM-1 that provides the interaction for the transmigration of monocytes across the epithelial cell model. More importantly, the inflammatory cytokine TNF-α released from ZIKV-infected macrophages affected the permeability of the BTB by degrading ZO-1 protein suggesting that IS can help ZIKV gain access to developing germ cells (46). Similar results were obtained with the use of a 3-D model of HBMECs. This experiment showed that apical junctions restrict the infection capability of several RNA viruses. However, treatment of the 3-D cultures with TNF-α disrupted the formation of cell-cell junctions and correlated with an increase in ZIKV viral RNA and infectious titers (51). Another study using RNAseq analysis of ZIKV-infected Sertoli cells showed that these cells responded to ZIKV with a diversity of innate antiviral mechanism. Importantly the analysis of upregulated genes on interferon, antigen presentation, and cross-talk between DC and NK signaling pathways correlated with the downregulation of genes involved in adherent junctions, tubulin α/β complex, and 14-3-3 signaling pathways (52). Interestingly, a clear correlation exists between the peak of the immune response and the downregulation of genes that control cell cycle progression. These data suggest that ZIKV could modulate the change from an immunosuppressive microenvironment in Sertoli cells to a strong antiviral response that alters the permeability of BTB with a concomitant delay in proliferation. This would prolong the time for viral progeny production, ensure persistence, and allow dissemination via sexual transmission.

Direct evidence of the disruption of cell junctions by ZIKV came from the use of the NS2A protein in mouse radial glial cells and human brain organoids. NS2A interacted directly with adherent junction components, such as ZO-1, β-catenin, SMAD7, and NUMBL, targeting them for lysosomal degradation and inducing aberrant cortical layer formation. We hypothesize that the same mechanism could be used by ZIKV in the testis to gain access to seminiferous tubules, but this needs to be tested (47).

**Figure 1:**
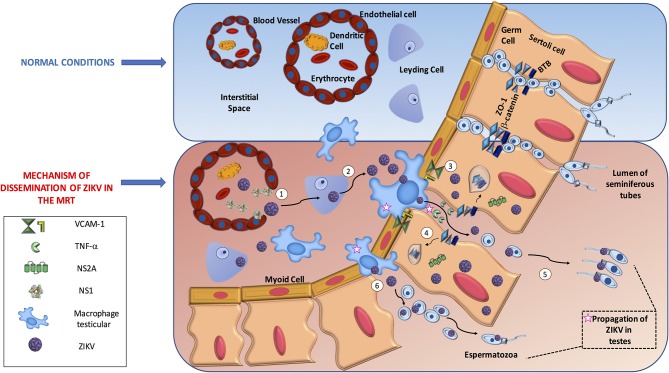
The dissemination process of ZIKV in male reproductive tract. In the male reproductive tract, Immune system mechanism might promote ZIKV dissemination. (1) ZIKV travel in infected immune cells such as Dendritic cells to testis; ZIKV NS1 protein increase the permeability of endothelial cells to allow virus access toward interstitial space, (2) ZIKV infect Leydig cells increase the production of viruses in the zone, (3) ZIKV can infect Sertoli cells and change the immunosuppressive microenvironment to a robust antiviral response. Activated macrophages secrete TNF-α that disrupt the Blood Testicular Barrier by degradation of tight junction proteins such as ZO-1 allowing ZIKV reach and infect germ cells, (4) In parallel, is possible that on infected Sertoli cells the NS2A protein target to degradation proteins of tight junction complexes helping also to reach germ cells, (5) Finally, through adhesion molecules such as VCAM-1 ZIKV infected macrophages can interact with Sertoli cells (6) and direct infiltrate the lumen of seminiferous tubules and ZIKV could infect eventually germinal cells.

Finally, evidence for the third mechanism was shown using 3-D culture HBMECs in transwell assays. ZIKV-infected monocytes crossed the epithelial barrier and infected primary human astrocytes plated in the basolateral chamber (51). Using a similar approach with Sertoli cells it was observed that ZIKV-infected macrophages interacted with and induced the permeability of the cells, suggesting that after induction the leakiness of the barrier allowed infiltration to the lumen of the seminiferous tubules and probably infection of germ cells (46). These studies suggest that the IS, when attempting to control viral infection, actually assists the entry of ZIKV to this immune-privileged site, causing persistence that allows sexual transmission (Figure 1).

On the other hand, within the human FRT, protection against sexually transmitted infection is opposed to biological fertility (53). The role of the FRT during ZIKV infection and sexual transmission has not been extensively evaluated. However, the mucosal immune system is the first line of defense against pathogens. The innate and adaptive elements of the mucosal immune system have evolved to meet the special challenges that are associated with the FRT (54). Interestingly, in contrast to that in males, the IS mechanism against ZIKV correlates with protection of the FRT. For example, interferon (IFN)-ε is constitutively expressed by epithelial cells in the FRT and protects against herpes simplex virus-2 infection. This protective role of the IFN-ε may also be prohibitive for ZIKV infection, but this needs to be tested (55). Protection via IFN response was shown by the administration of recombinant IFN-β/λ in primary human vaginal/cervical epithelial cells. This restricted ZIKV replication by induction of canonical IFN-stimulated genes (56). In agreement with this study, the induction of a strong IFN response is necessary to prevent vaginal ZIKV infection in mice because the virus elicits minimal induction of IFN response and seems to regulate the activation of antigen presenting cells (57).

**Figure 2:**
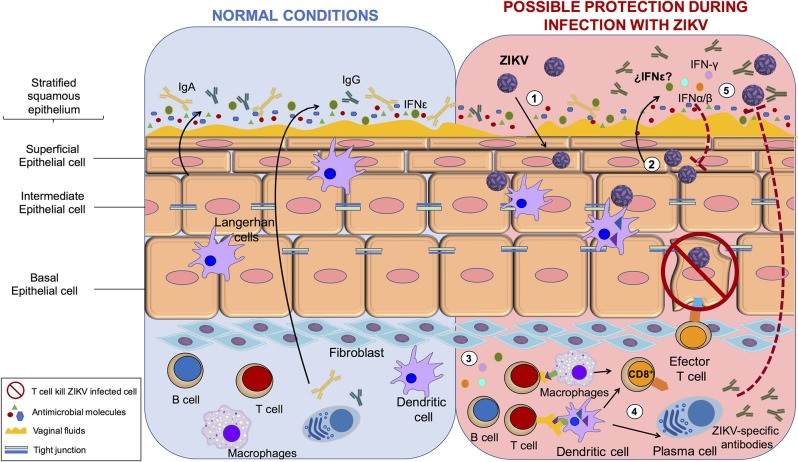
The protection process of ZIKV in female reproductive tract. Immune system mechanism protects against ZIKV infection in female reproductive tract. (1) ZIKV infect vaginal epithelial cells, (2) infected cells produce IFN-α,β,γ, and possibly IFN-ε which initiates the intracellular antiviral state (3) immune cells increases antigen presentation leading to T and B cell activation, (4) effector B cells secrete ZIKV-specific antibodies and effector cytotoxic T cells kill ZIKV infected cells; (5) Finally, the IS mechanism control and protect against ZIKV.

Additionally, it is necessary to consider the regulation of the immune system by sex hormones when evaluating ZIKV infection of the FRT. For example, estradiol and progesterone coordinate unique patterns of epithelial cells, stromal fibroblast, and immune cell functions that optimize the conditions for fetal survival and maternal protection (58). This is supported by the analysis of an immunocompetent animal model infected with ZIKV. Hormonal treatments in the FRT promoted the transmission and replication of virus. This study suggested that women establish correct innate and cellular antiviral mechanisms and hormonal regulation to control the infection (59). In mice, subcutaneous ZIKV inoculation elicited a cellular and humoral response that protected subsequent intravaginal inoculation (60). These studies suggest that in females a robust immune response protects against sexual exposure instead of helping the viral establishment and persistence. In conclusion, these data show that antiviral IS mechanisms in the FRT correlate with protection, the opposite of the response mechanism in the MRT. This is in agreement with clinical data indicating that the female-to-male route of sexual transmission is less frequent than the male-to-female route is. However, is important to consider other factors associated with the sexual transmission of the ZIKV. For example, it was reported that the microtrauma in the epithelial barrier of FTR during intercourse promotes the sexual transmission of HIV-1 or HVB (61, 62). In addition, relevant future studies are needed to understand the role of hormones that regulated the menstrual cycle and the effect on sexual transmission of ZIKV in the FRT. Recently, it has been suggested that estradiol is associated with activation of the innate immune mechanism and initiation of the antiviral responses, and that the modulation by sexual hormones results in alteration of a woman's susceptibility to HIV-1 and other infections (61, 63). The understanding about the role of hormones in different stages of menstrual cycle or pregnancy may help to resolve question on the mechanism of ZIKV to persist in the FRT (Figure 2).

Finally, whether sexual transmission (STx) has any influence on vertical transmission (Vtx) of ZIKV is yet unanswered. Until now, the literature shows that the mechanisms of immune response in FRT can control ZIKV infection via STx, and in humans, to our knowledge, data indicating Vtx after sexual transmission does not exist. However, a mouse model with suppressed IFN response showed that males have infectious viruses in the testicles and can transmit sexually to females during the early phase of infection. Importantly, in the few cases of pregnancy with STx of ZIKV, there was only evidence of infection in cells of the uterus and placenta, but there was no clear evidence of Vtx in the fetuses. In contrast, Vtx was clear after peripheral ZIKV infection, since 26% of brains of the fetuses/pups were positive for ZIKV RNA (64).

In the context of peripheral infection, the mechanisms described to alter BTB could be used by ZIKV to infect the placenta. A study shows that placental macrophages can be infected by ZIKV and respond to the infection by inflammatory and antiviral signaling pathways. The authors propose that placental macrophages support efficient ZIKV replication, and this may result in the subsequent infection of neural progenitor cells (65). However, a report showing Vtx without breakdown of the placental barrier also exists. The discordance in these studies show the complexity of the ZIKV infection and argue that these mechanisms may have a role during the Vtx, but more studies are needed to evaluate them correctly.

## Conclusions

ZIKV can infect and regulate different cells, including immune system cells, to ensure survival and persistence. In the MRT, the activation of different immunological events in response to ZIKV infection correlate with viral persistence that, conversely, in females, are necessary to inhibit the virus and control the infection. We highlight some important aspects of the immune system that assist ZIKV dissemination into the reproductive organs, thus showing that IS can have a dual role in the pathogenesis of sexual transmission. However, the molecular mechanism used by ZIKV in human cells to guarantee infection has not been investigated thoroughly. Understanding the molecular mechanisms utilized by the virus for dissemination will be invaluable to develop new targets for therapeutic intervention.

## Author Contributions

ML and HA designed the concept and completed the final editing of the manuscript. All authors contributed to writing of the manuscript. MV and TV prepared the figures. All authors read approved the manuscript for publication.

### Conflict of Interest Statement

The authors declare that the research was conducted in the absence of any commercial or financial relationships that could be construed as a potential conflict of interest.
